# Supplementary material for: Comprehensive Annotation and Functional Exploration of MicroRNAs in Lettuce
Source: Front Plant Sci. 2021 Dec 24;12:781836. doi: 10.3389/fpls.2021.781836 (PMC8739914; doi:10.3389/fpls.2021.781836)
Supplement: Supplementary file 3 [file Data_Sheet_9.ZIP › GSM2230751.plot/Lsa-miR394b_Lsat_1_v5_gn_1_87300.1_6292_TPlot.pdf]

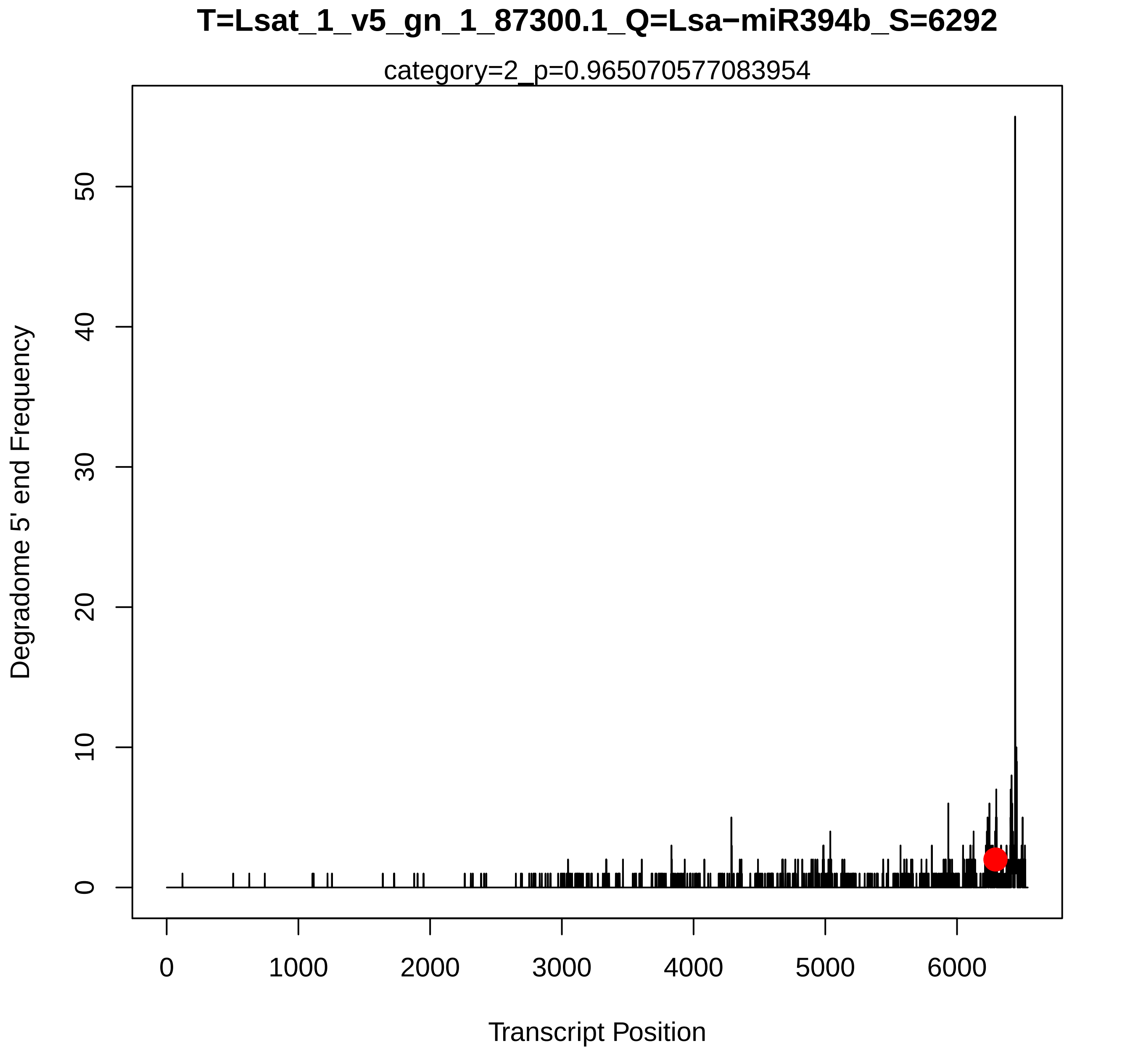
T=Lsat_1_v5_gn_1_87300.1_Q=Lsa−miR394b_S=6292
category=2_p=0.965070577083954
Degradome 5' end Frequency
0
10
20
30
40
50
0
1000
2000
3000
4000
5000
6000
Transcript Position
